# Synthesis and Characterization of Oil-Chitosan Composite Spheres

**DOI:** 10.3390/molecules18055749

**Published:** 2013-05-16

**Authors:** Keng-Shiang Huang, Chih-Yu Wang, Chih-Hui Yang, Alexandru Mihai Grumezescu, Yung-Sheng Lin, Chao-Pin Kung, I-Yin Lin, Yi-Ching Chang, Wei-Jie Weng, Wei-Ting Wang

**Affiliations:** 1 The School of Chinese Medicine for Post-Baccalaureate, I-Shou University, Kaohsiung 82445, Taiwan; E-Mails: huangks@isu.edu.tw (K.-S.H.); vina920715@hotmail.com (C.-P.K.); amy71756@hotmail.com (I.-Y.L.); ballballadult@hotmail.com (Y.-C.C.); merrill92612@hotmail.com (W.-J.W.); sheep8068@hotmail.com (W.-T.W.); 2 Department of Biomedical Engineering, I-Shou University, Kaohsiung 82445, Taiwan; 3 Department of Biological Science and Technology, I-Shou University, Kaohsiung 82445, Taiwan; E-Mail: chyang@isu.edu.tw; 4 Faculty of Applied Chemistry and Materials Science, Department of Science and Engineering of Oxidic Materials and Nanomaterials, University Politehnica of Bucharest, Bucharest 011061, Romania; E-Mail: grumezescu@yahoo.com; 5 Department of Applied Cosmetology and Master Program of Cosmetic Science, Hungkuang University, Taichung 43302, Taiwan; E-Mail: linys@sunrise.hk.edu.tw

**Keywords:** oil-chitosan composite spheres, iron oxide, dual encapsulation

## Abstract

Oil-chitosan composite spheres were synthesized by encapsulation of sunflower seed oil in chitosan droplets, dropping into NaOH solution and *in situ* solidification. Hydrophilic materials (*i.e.*, iron oxide nanoparticles) and lipophilic materials (*i.e.*, rhodamine B or epirubicin) could be encapsulated simultaneously in the spheres in a one step process. The diameters of the prepared spheres were 2.48 ± 0.11 mm (pure chitosan spheres), 2.31 ± 0.08 mm (oil-chitosan composites), 1.49 ± 0.15 mm (iron-oxide embedded oil-chitosan composites), and 1.69 ± 0.1 mm (epirubicin and iron oxide encapsulated oil-chitosan composites), respectively. Due to their superparamagnetic properties, the iron-oxide embedded oil-chitosan composites could be guided by a magnet. A lipophilic drug (epirubicin) could be loaded in the spheres with encapsulation rate measured to be 72.25%. The lipophilic fluorescent dye rhodamine B was also loadable in the spheres with red fluorescence being observed under a fluorescence microscope. We have developed a novel approach to an *in situ* process for fabricating oil-chitosan composite spheres with dual encapsulation properties, which are potential multifunctional drug carriers.

## 1. Introduction

Chitosan is a natural polysaccharide that has several advantages, including non-toxicity, biocompatibility and biodegradability. Therefore, it is useful for interdisciplinary applications, such as controlled drug release, drug carriers, gene delivery, tissue engineering, as a plant growth and cell metabolism, hemostatic agent, “fat binder”, and for the filtration of phosphorus, heavy minerals, oils, and others hazardous materials [1,2,3,4,5,6].

Magnetic-responsive chitosan spheres are a combination of two materials (*i.e.*, chitosan and iron oxide) that form multifunctional particles which allow for more applications than particles with a single ingredient [7,8,9]. Magnetic composite particles were extensively discussed in the literature, including Fe_3_O_4_ nanoparticles-chitosan composite particles [5,7,9,10,11]. The combinations of iron oxide nanoparticles and chitosan matrices exhibit good mechanical and functional properties and can be applied in various fields, such as recyclable responsive drug release, magnetic resonance imaging (MRI) enhancement, heavy metal removal, *etc.* [11,12,13,14]. Recently, chitosan-based amphiphilic drug-carriers have attracted the attention of several research groups. Amphiphilic chitosan spheres with a hydrophobic core and a hydrophilic shell can be used as carriers for hydrophobic and hydrophilic drugs simultaneously [15]. Kim *et al.* employed hydrophobic cholanic acid to modify glycol chitosan to form amphiphilic nanoparticles by self-assembly in water solution [16]. In the literature, a new type of amphiphilic chitosan, namely carboxymethylhexanoyl chitosan (CHC) was successfully synthesized [17,18]. Asthanaa *et al.* adopted nanoemulsion template-based chitosan nanocapsules to encapsulate amphotericin B. The amphiphilic properties are ensured by the inclusion of an oil phase [19].

Due to its natural non-toxicity and biocompatibility properties, chitosan has become a good material for encapsulation of some environmentally sensitive ingredients such as lipophilic drugs and vitamins, enzymes, antigens, olive oil extract, *etc.* [20,21,22,23,24,25,26,27,28]. The encapsulation of oil by chitosan deserved mention because it suggests the potential for use as a drug carrier that can encapsulate both hydrophilic and lipophilic materials simultaneously, but this was little discussed. Several related research topics can be found in the food industry. Klaypradit *et al.* developed a technique to encapsulate tuna oil in chitosan by using ultrasonic atomization and freeze drying [29]. Klinkesorn *et al.* adopted lecithin-chitosan membranes to stabilize spray-dried tuna oil-in-water emulsions, which has potential as an ω-3 fatty acid ingredient for functional foods due to its increased oxidative stability compared to bulk oils [30]. For controlled release of volatile materials, Hsieh *et al.* encapsulated citronella oil with heat-shrinking chitosan microcapsules, which possess pore spaces between the wall membrane molecules that are changeable under heat treatment to achieve the controlled release effect [31]. 

**Figure 1:**
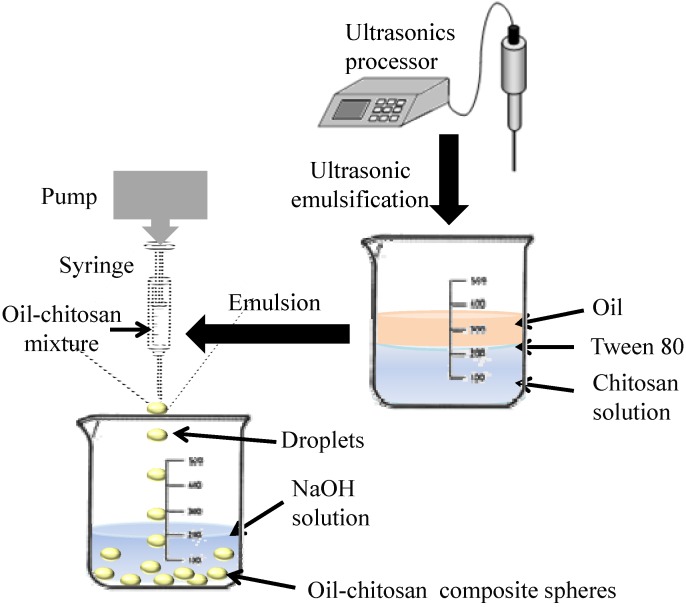
Schematic of the preparation of oil-chitosan spheres.

In this study, we present a facile approach to fabricate oil-chitosan composite particles through encapsulation of sunflower seed oil in chitosan spheres. Hydrophilic materials (*i.e.*, iron oxide nanoparticles) and lipophilic materials (*i.e.*,rhodamine B or epirubicin) could be encapsulated simultaneously in the spheres. Figure 1 shows the one step process for production of the superparamagnetic oil-chitosan composite spheres through *in-situ* co-precipitation and the gelation of ferro-gels. Due to its abundant amino groups, chitosan has a good capability for the uptake of ferrous and ferric cations via chelation or ion exchange mechanisms [32]. Ferrous and ferric cations were mixed with an oil-chitosan emulsion to form the ferro-gel solution. Solidification was subsequently achieved by dropping the ferro-gel droplets into a NaOH solution to form the oil-chitosan spheres with embedded iron oxide nanoparticles. In contrast to the spray-drying or freeze drying methods, this process prevents oil changes due to exceedingly high or low temperatures. One step synthesis of multifunctional capsules is another significant advantage of the present approach.

## 2. Results and Discussion

### 2.1. Morphology

**Figure 2:**
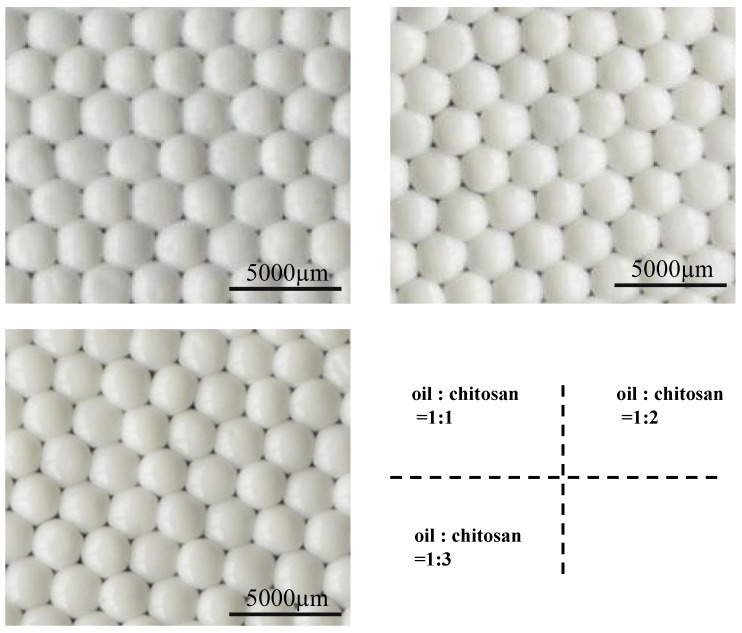
The oil-chitosan composite sph eres prepared by using various volume ratio (the ratios are 1:1, 1:2 and 1:3, respectively) and solidified in 20% NaOH solution.

Figure 2 shows the oil-chitosan composite spheres obtained from various oil-chitosan ratios. Different from the color of pure chitosan spheres (which looked somewhat transparent), the fabricated spheres looked milky white. This may be due to the participation of the oil. The appearances of the spheres showed no obvious differences among the three kinds of oil-chitosan ratios tested; however, the spheres became more non-friable with higher oil-chitosan ratio. Therefore we adopted the spheres fabricated with an oil-chitosan ratio of 1:3 for subsequent study. 

**Figure 3:**
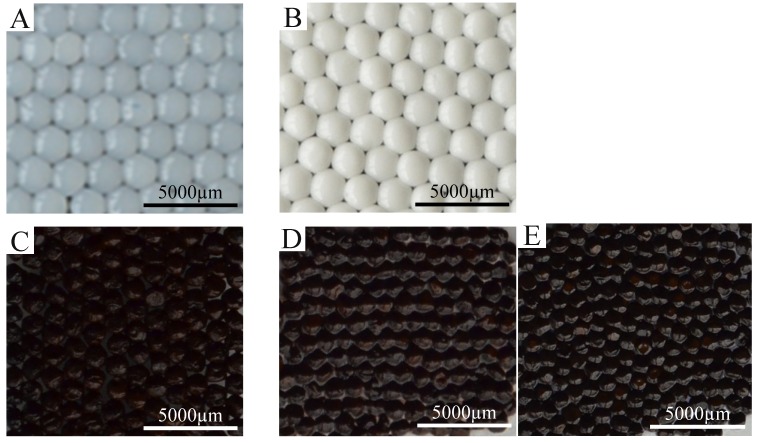
Spheres prepared in this study. (**A**) Pure chitosan spheres. (**B**) Oil-chitosan spheres. (**C**) Iron oxide nanoparticles embedded oil-chitosan spheres. (**D**) Encapsulated epirubicin-embedded iron oxide nanoparticle oil-chitosan spheres. (**E**) Encapsulated rhodamine B-embedded iron oxide nanoparticle oil-chitosan spheres. Synthesis conditions are described as follows: the volume ratio of chitosan solution:oil:iron ion solution is 3:1:2. The dose of epirubicin is 0.2 mg/10 mL. NaOH solution (20 wt %) was used for solidification.

Figure 3 shows the morphological characteristics of the prepared particles. Figure 3A shows a photograph of the pure chitosan spheres (with an average diameter of 2.48 ± 0.11 mm). Figure 3B shows a photograph of the oil-chitosan spheres (with an average diameter of 2.31 ± 0.08 mm). Figure 3C shows a photograph of the iron-oxide embedded oil-chitosan composites spheres (with an average diameter of 1.49 ± 0.15 mm). Figure 3D shows a photograph of the epirubicin and iron oxide encapsulated oil-chitosan composites spheres (with an average diameter of 1.69 ± 0.1 mm). There were no significant changes between pure chitosan spheres and oil-chitosan composite spheres (Figure 3A *vs* Figure 3B). Comparing the size of the oil-chitosan composite spheres between that with and without encapsulated iron oxide (Figure 3B,C), however, we found that the latter was much smaller than the former (by about 60%). This may be due to the co-precipitation effects of ferro-gels and the chitosan molecules. The sizes of iron-oxide embedded oil-chitosan composites spheres with and without epirubicin are almost the same (Figure 3C,D).

In the future, the diameters of the spheres could be made smaller by employing other conventional droplet generation methods, such as atomization (spraying), emulsification, coacervation, sonication, electrostatic droplets, microfluidic droplets, *etc.* [2,11,33,34,35,36,37]. In Figure 3C,D it can be seen that the iron-oxide embedded spheres were black, indicating the presence of the iron-oxide nanoparticles.

Yang *et al.* presented a synthesis of superparamagnetic chitosan spheres with a macroporous internal structure. Using a one step process, the spheres could be easily prepared [5], but the sphere size is not easily controlled. For example, smaller spheres should employ smaller needles, which are not commercially available and must be hand-made or customized.

In the present study, we report the fabrication of oil-chitosan spheres using microfluidic technology. This approach has several advantages such as sphere size controllability, monodispersity, and high throughput. In addition, the prepared oil-chitosan spheres could encapsulate hydrophilic materials and lipophilic materials simultaneously in the same sphere.

### 2.2. Characterization

**Figure 4:**
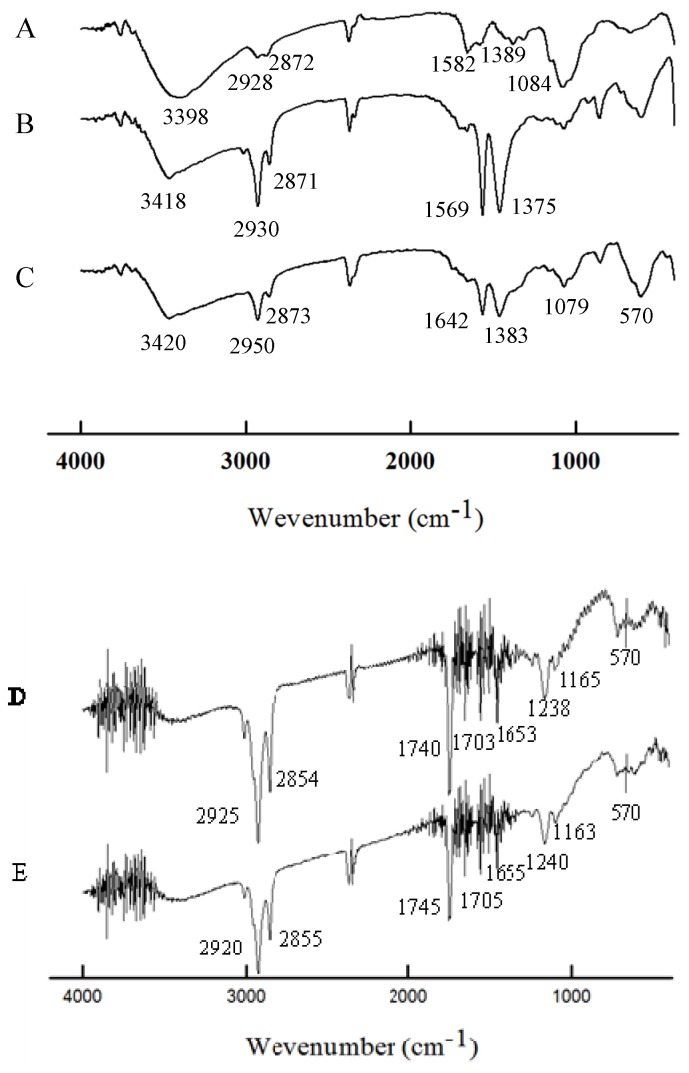
FTIR spectra of pure chitosan spheres (curve **A**), oil-chitosan spheres (curve B), iron-oxide embedded oil-chitosan composite spheres (curve **C**), Rhadamine B and iron oxide encapsulated oil-chitosan composite spheres (curve **D**), Epirubicin and iron oxide encapsulated oil-chitosan composite spheres (curve **E**).

Figure 4 shows the FTIR spectra of the pure chitosan spheres, oil-chitosan composite spheres, embedded iron oxide-oil-chitosan composite spheres, rhodamine B loaded - embedded iron oxide oil-chitosan spheres, and epirubicin loaded - embedded iron oxide oil-chitosan spheres, respectively. The peaks around 3398–3420 cm^−1^ relate to the –OH group of adsorbed water (curves **A**, **B** and **C**). In the spectrum of chitosan (curve **A**), the characteristic absorption peaks appeared around 2,928 cm^−1^ and 2,872 cm^−1^ (attributed to the C–H groups of the backbone polymer), 1,582 cm^−1^ (attributed to the N-H bending vibration) and 1,389 cm^−1^ (attributed to -C-O stretching of the primary alcoholic groups in chitosan). The characteristic peaks have somewhat shifted in curves B to D, but are still present, indicating the presence of chitosan. The shifts indicate that chitosan reacts with glutaraldehyde to form a Schiff base [9,10]. In the spectrum of the oil-chitosan composite spheres (curve **B**), the characteristic peaks of sunflower seed oil appeared at 2,871 to 2,950 cm^−1^ (attributed to symmetric and asymmetric stretching vibrations of the aliphatic CH_2 _and CH_3 _groups) [38,39]. Similar peaks were visible in curves **C** and **D**. Observing the spectrum of the iron oxide-loaded oil-chitosan composite spheres (curve **C**), we find that a characteristic peak appears around at about 570 cm^−1^ (attributed to the Fe–O group), indicating that iron oxide nanoparticles were successfully embedded in the chitosan particles. The results suggested that the synthesized spheres should have superparamagnetic properties because of the Fe_3_O_4_ nanoparticles. In curves **D** and **E**, there were absorption peaks at 2,854 cm^−1^ to 2,925 cm^−1^, 1,163 cm^−1^ to 1,240 cm^−1^ and a group of peaks (1,653 cm^−1^ to 1,745 cm^−1^), indicating the absorptions of rhodamine B or epirubicin [40,41].

### 2.3. Magnetic Response and Dual Encapsulation Properties

**Figure 5:**
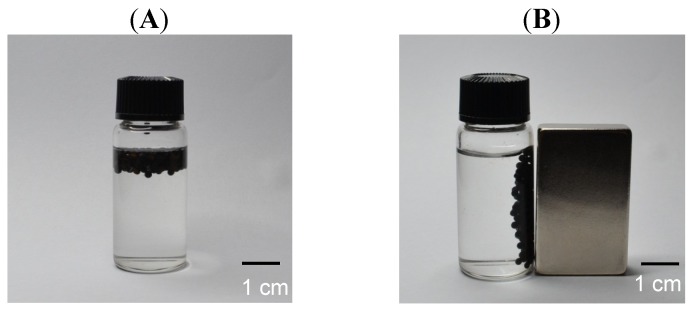
Iron oxide nanoparticle-loaded chitosan spheres were attracted to wall of the vial using an external magnetic field.

The magnetic responsive property was tested with an external magnet. The Fe_3_O_4_ nanoparticle-loaded oil-chitosan composite spheres were floating in water (Figure 5A). We presume that the spheres have less specific gravity than water due to fact they contain oil. These spheres can be attracted and drawn through the solution to the side wall of the vial by an externally applied magnetic field (Figure 5B), indicating that these spheres have superparamagnetic properties.

**Figure 6:**
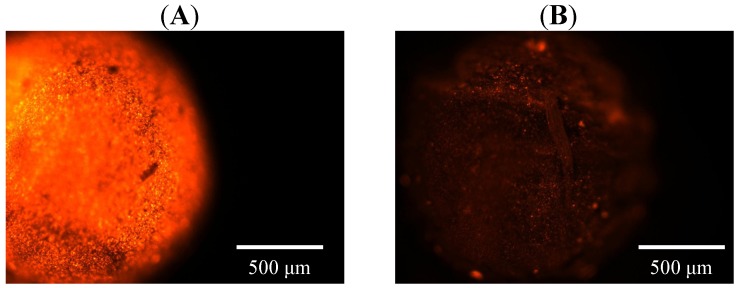
Fluorescence images of encapsulated rhodamine B—embedded iron oxide nanoparticle oil-chitosan spheres obtained with a fluorescent microscope. (**A**) Fluorescence images at the beginning of photo capture. (**B**) Fluorescence images 10 minutes later. The fluorescence intensity had seriously decayed.

In addition to the encapsulation of hydrophilic materials (*i.e.*, iron oxide nanoparticles), lipophilic materials (*i.e.*, rhodamine B or epirubicin) could also be loaded in the spheres. Figure 6 shows the fluorescence microscopy image of encapsulated rhodamine B iron oxide nanoparticle-embedded oil-chitosan spheres. The red fluorescence on the spheres indicated that rhodamine B has been successfully loaded in the spheres (Figure 6A). In Figure 6B, fluorescence was quenched 10 minutes later. Furthermore, epirubicin could be encapsulated in the oil-chitosan composite spheres with an encapsulation rate measured to be 72.25%. These results indicated dual encapsulation properties (the capability to encapsulate hydrophilic and lipophilic materials simultaneously) of the fabricated spheres.

## 3. Experimental

### 3.1. Materials

Chitosan (molecular weight: 150,000), iron(II) chloride tetrahydrate (FeCl_2_•4H_2_O, 99%), iron (III) chloride hexahydrate (FeCl_3_•6H_2_O, 98%), sodium hydroxide (NaOH), acetic acid (CH_3_COOH) and Tween 80 were purchased from Sigma-Aldrich, J. T. Baker, Alfa Aesar, Mallinckrodt, and Nihon Shiyaku Reagent, respectively, and used as received without further processing. Sunflower seed oil was obtained from Uni-President Enterprise Co. (Tainan, Taiwan).

### 3.2. Synthesis of Chitosan Spheres

Chitosan (0.04 g, dissolved in 2 mL of 1%, v/v CH_3_COOH solution) was dropped into a NaOH solution (20 wt %) by means of a syringe and pump. After 10 min, chitosan spheres were observed. Particles were collected by centrifugation and washed twice with dd-H_2_O (30 mL) to remove any alkali.

### 3.3. Synthesis of Oil-Chitosan Composite Spheres

Chitosan (0.04 g, dissolved in 2 mL of 1%, v/v CH_3_COOH solution) and sunflower seed oil (in a chitosan to oil ratio of 3:1) were mixed through constant stirring for 10 min. The solution was then dropped into a NaOH solution (20wt %), by means of a syringe and pump. After 10 minutes, encapsulated oil-chitosan microparticles were observed. Particles were collected by centrifugation and washed twice with dd-H_2_O (30 mL) to remove any alkali.

### 3.4. Synthesis of Superparamagnetic Oil-Chitosan Composite Spheres

Chitosan (0.04 g, dissolved in 2 mL of 1%, v/v CH_3_COOH solution), sunflower seed oil (in a chitosan to oil ratio of 3:1), FeCl_2_•4H_2_O (0.199 g, dissolved in 0.5 mL of 2N HCl solution) and FeCl_3_•6H_2_O (0.135 g, dissolved in 0.5 mL of 2N HCl solution) were mixed through constant stirring for 10 minutes to obtain the ferro-gel solution, which was then dropped into NaOH solution (20 wt %) by means of a syringe and pump. After 10 minutes, Fe_3_O_4_-oil-chitosan composite spheres having a black color were observed. Spheres were collected by centrifugation and washed twice with dd-H_2_O (30 mL) to remove any alkali.

### 3.5. Preparation of Epirubicin and Encapsulated Iron Oxide Oil-Chitosan Composites

Epirubicin (0.2 g) was dissolved in iron ion-oil-chitosan solution (10 mL, ingredients were as described in the previous section), and then dropped into NaOH solution (20 wt %) by means of a syringe and pump. After 10 minutes, epirubicin-Fe_3_O_4_-oil-chitosan composite spheres having a black color were observed. The fabricated spheres were collected by centrifugation and washed twice with dd-water (30 mL) to remove any residual epirubicin and alkali.

### 3.6. Instruments

Size distributions of the various droplets samples were obtained from the random sampling of about 50 individual spheres so as to minimize selection bias. An inverted microscope system, including an optical microscope (BX60, Olympus, Tokyo, Japan) and a digital camera (DP70, Olympus), were employed for imaging. The average diameter of the spheres, expressed as mean ± standard deviation, was obtained from the photomicrographs. A Vibra-Cell Ultrasonics Processor (VCX130, Sonics & Materials, Inc., Newtown, CT, USA) was used for emulsification to obtain oil-chitosan mixtures. A High Performance 2UVTM Transilluminator (LM-20E, Krackeler Scientific, Inc., Albany, NY, USA) was employed to observe the fluorescence of the rhodamine B loaded spheres. Fourier transform infrared spectroscopy (FTIR) spectra were recorded with a Spectrum One FTIR spectrometer (Perkin Elmer, Waltham, MA, USA), using KBr pellets, in the range of 400–4,000 cm^−1^, with a resolution of 4 cm^−1^.

## 4. Conclusions

We propose a one-step approach for the manufacture of oil-chitosan composite spheres that can encapsulate simultaneously hydrophilic materials and lipophilic materials. The diameters of the fabricated spheres were about 1.5 mm to 2.5 mm, but it should be possible to significantly reduce the particle size in the future by employing electrostatic or microfluidic droplet technology. Magnetic responsive activity was shown by attracting and dragging the spheres to the side wall of a vial through application of an external magnetic field using a simple magnet. Rhodamine B and epirubicin were used to verify the capability of encapsulating lipophilic drugs. The results suggest that the prepared oil-chitosan composite spheres have potential for use as dual encapsulation drug carriers in multidisciplinary applications.
